# Optical coherence tomography angiography of central serous chorioretinopathy: quantitative evaluation of the vascular pattern and capillary flow density

**DOI:** 10.1007/s00417-021-05306-w

**Published:** 2021-09-10

**Authors:** Farci Roberta, Carta Arturo, Fossarello Maurizio

**Affiliations:** 1 Eye Clinic, Department of Surgical Sciences, University of Cagliari, Via Ospedale, 46, 09124 Cagliari, CA Italy; 2 Department of Health Sciences, Università Degli Studi Di Milano, Milan, Italy; 3 Ophthalmology Unit, Department of Medicine and Surgery, University Hospital of Parma, Via Gramsci 14, 43126 Parma, Italy

**Keywords:** Central serous chorioretinopathy, OCT angiography, Choroidal capillary layer, Choroidal patterns

## Abstract

**Background:**

This study aimed to evaluate the vascular pattern and capillary flow density (CFD) map on optical coherence tomography angiography (OCTA) images of patients affected by central serous chorioretinopathy (CSC).

**Methods:**

In this retrospective cohort study, OCTA (AngioVue RTVue XR Avanti, Optovue) 3 × 3 mm macula scans of both eyes of patients with CSC were taken at baseline; the images were segmented and compared with OCTA scans of fellow eyes without CSC as well as age-matched healthy subjects. OCTA images were processed by quantitative textural analysis (ImageJ software) to provide an objective grading of choroidal capillary alterations. The texture of OCTA images was examined by the autocorrelation method.

**Results:**

In eyes with CSC (40 eyes), we found six different morphological patterns of the choriocapillaris layer vasculature (CCL), likely corresponding to different grades of OCT choroidal hyporeflectivity and OCTA reduction of the decorrelation signal. Moreover, the OCTA capillary flow density map revealed capillary depletion in the superficial capillary plexus (p value = 0.0023), in the deep vascular network (p value =  < 0.0001), and in the CCL (p value = 0.0001). Such findings were not observed in healthy subjects (13 eyes).

**Conclusions:**

OCTA in CSC is a useful tool that allows the identification of the clinical type of CSC by means of specific CCL patterns. Moreover, CFD depletion is observed in association with the inner retinal layers, pointing to an involvement of the inner blood retinal barrier in CSC. According to our results, it is plausible that the patterns observed herein may correlate to the different clinical subtypes of the disease. 
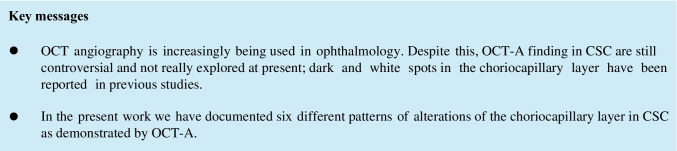

## Introduction

Central serous chorioretinopathy (CSC) is characterised by serous neuroretinal detachment most commonly involving the macular region and often involving the retinal pigment epithelium (RPE). Described over 150 years ago by Albrecht von Graefe, CSC is the fourth most common cause of retinopathy, with a reported incidence of 9.9 and 1.7 per 100,000 in men and women, respectively; it usually occurs in young males but may be observed in older subjects of both genders [1]. The pathogenesis of CSC has yet to be fully clarified. Alterations in choroidal circulation are probably the main mechanism leading to the development of CSC [2]. Tewari et al.[3] suggested that autonomic dysfunction (increased sympathetic and decreased parasympathetic activities) may lead to the inability of choroidal vessels to maintain homeostasis; this condition eventually may cause choroidal hyperperfusion and venous congestion with an increase in choroid hydrostatic pressure and choroidal permeability, ultimately resulting in subretinal fluid (SRF) accumulation [4, 5].

Although a consensual definition of the various clinical subtypes of CSC and their exact boundaries does not exist, in general, the clinical course of the disease can be classified as one of three main types: acute, chronic, or recurrent [6]. In most cases, the natural history of CSC shows a self-limiting course, but some patients are known to present with persistent, recurrent, or even bilateral CSC with distressing visual loss. In the acute phase of CSC, the neurosensory retina may be thickened within the area of serous retinal detachment [7]. In chronic CSC, intraretinal cysts or schisis can persist, causing diffuse retinal pigment epitheliopathy[8] or choroidal neovascular membrane formation [9], associated with permanent loss of central vision [10]. More recently, CSC has been described as a part of the spectrum of pachychoroid disease, which includes pachychoroid pigment epitheliopathy, CSC, pachychoroid neovasculopathy, and polypoidal choroidal vasculopathy (PCV) [11].

Spectral domain optical coherence tomography (SD-OCT) is a relatively novel imaging technique that has uncovered a number of previously unappreciated features in CSC eyes, such as intraretinal fluid accumulation [8], outer nuclear segment thickening and elongation [12], cystoid macular degeneration [9], loss of inner segment/outer segment integrity [13], and an increase in the choroidal thicknesses of both the affected and contralateral eyes (mirroring the hyperpermeability of the choriocapillaris) [14].

Optical coherence tomography angiography (OCTA) in ophthalmology is a noninvasive, depth-resolved imaging technique based on the concept of detection of changes in blood flow, which allows for the examination of the retinal and choroid vasculature without requiring any dye injection [15, 16].

OCTA imaging of CSC has permitted us to better delineate some aspects of the choroidal circulation, such as abnormal choriocapillaris and choroid flow patterns [17, 18], not only in CSC eyes but also in fellow eyes without serous retinal detachment [19], and dark areas and dark spots observed in the choriocapillaris [17, 18], which have been interpreted as possible focal flow reductions that should be distinguished from possible imaging artefacts [19, 20]. Moreover, inner retinal microvasculature alterations in the macular region have been described [21].

The aim of our work was to study the vascular pattern and capillary flow density (CFD) map on OCTA images of CSC patients and healthy controls and to evaluate specific changes related to the clinical type of CSC.

## Materials and methods

This study was a single-institution observational case–control series of consecutive patients with CSC seen at the outpatient unit of the University Eye Clinic of Cagliari, Italy, from December 2014 to June 2016. We included patients with a clinical history of CSC and visual impairment, treatment-naive patients, and newly diagnosed CSC patients. To be included as a case, each patient had to present the clinical hallmark of CSC according to what was reported in the most recent literature, i.e. the presence of a serous detachment of the neurosensory retina limited to the macular region may sometimes be associated with a serous RPE detachment (leakage of fluid through the RPE into the subretinal space) as documented by appropriate ocular imaging; we defined “chronicity” of the disease as the persistence of subretinal fluid for at least 6 months. OCTA images of thirteen sex- and age-matched normal individuals were selected as controls. All the patients gave their informed consent to undergo all the examinations required by our medical staff. Ethical approval for retrospective analysis of this observational case series was granted by the Institutional Review Board at Cagliari University, and the study adhered to the tenets of the Declaration of Helsinki. Patients were identified via a review of electronic medical records. Relevant demographic data, including age, sex, race, and ocular comorbidities, were collected. Exclusion criteria included age > 55 years, myopia > 6 dioptres, evidence of choroidal neovascularization, previous medical or laser treatments for CSC, smoking, blood hypertension, cardiovascular disease, alterations in the plasma lipid profile, diabetes, arteriosclerosis, other ocular comorbidities, or surgery. There were 39 patients who met the inclusion criteria; 40 eyes were evaluated with fundus multimodal imaging, including OCTA. All selected patients underwent a complete ophthalmic examination by a retina specialist (FR), and all eyes included were examined with dilated-fundus biomicroscopy, infrared and autofluorescence (AF) (Spectralis, Heidelberg, Germany), fluorescein angiography (FA) (Topcon, Germany), indocyanine green angiography (ICGA) (Spectralis, Heidelberg, Germany), SD-OCT (XR Avanti; Optovue, Inc., Fremont, CA, USA), and OCTA using RTVue XR Avanti with angioVue^R^ (Optovue, Freemont, CA, USA). This OCTA platform incorporates split-spectrum amplitude decorrelation angiography (SSADA) software algorithm generating 3-dimensional en face angiograms through decorrelation of two merged consecutive orthogonal registration volumes automatically centred on the macula or manually centred on the lesion, and a CFD map software as a part of the routine CSC fundus evaluation.

Each image was evaluated by assessing the absence of RPE detachment or irregularities on the AF, on the OCT B-Scan and en face frames obtained with Cirrus 4000, and on the en face realised by the Optovue device; imaging with window defects and pooling on fluorescein and indocyanine angiography were excluded from this study.

The RTVue XR Avanti (Optovue, Inc.) provides amplitude-decorrelation angiography images. It has an A-scan rate of 70,000 scans per second, using a light source centred at 840 nm (bandwidth: 50 nm). Each OCTA volume contained 304 × 304 A-scans, with two consecutive B-scans captured at each fixed position before proceeding to the next scan. The OCTA volume acquisition time is approximately 3 s; the split-spectrum mode using two orthogonal volumes provides the motion correction necessary for minimising artefacts due to microsaccades and eye movement.

Optovue segmentation includes the superficial capillary plexus (SCP), deep capillary plexus (DCP), outer retinal layer (ORL), and choriocapillaris plexus (CCL). Since ORL in our OCTA images was avascular, we used only images of the three vascular plexuses, as obtained with automated segmentation, for analysis of the capillary flow density (CFD). To measure the capillary flow density (CFD), we used internal map software (version 2015.100.0.35 AngioAnalytics, Optovue, Inc.), which measures the CFD as a grid-based percentage of the sample area occupied by vessel lumens, after intensity thresholding and image segmentation; the choroidal layer was automatically segmented.

In general, OCTA imaging allows us to obtain percentage values of the CFD in four predetermined sectors using a colour scale. The macular region is subdivided into 18 subregions, and a percentage value is calculated for each zone.

Two retina specialists (FR and FM) acquired the images and performed a qualitative analysis to determine CSC features. Although there is currently no universally accepted classification system for CSC, we classified our patients according to the following main types [6]:

- Acute CSC: patients with spontaneous complete resolution of subretinal fluid (SRF) in 3–6 months;
- Recurrent CSC: patients who had previous episodes of CSC after complete SRF resolution;
- Chronic CSC: patients with persistent serous detachment(s) as seen on OCT scans for longer than 6 months.

To assess the microvascular architecture, one trained OCTA user (FR) examined 3 × 3 mm scanning areas at the three depths previously indicated (i.e. SCP, DCP, CCL), as obtained with automated segmentation, and quantified by OCT software on the basis of the distance between the inner limiting membrane (ILM) and retinal pigment epithelium (RPE).

CCL vascular patterns were identified on the basis of the extension of dark areas, as detected using ImageJ software (https://imagej.nih.gov/NIH, Bethesda, MD, USA). Eight-bit grey OCTA images were cropped from the original OCTA images to remove small graphical symbols near the image border. The final images were 512 × 512 pixels, with each pixel corresponding to 5 μm. These images were then evaluated via textural analysis.

**Fig. 1:**
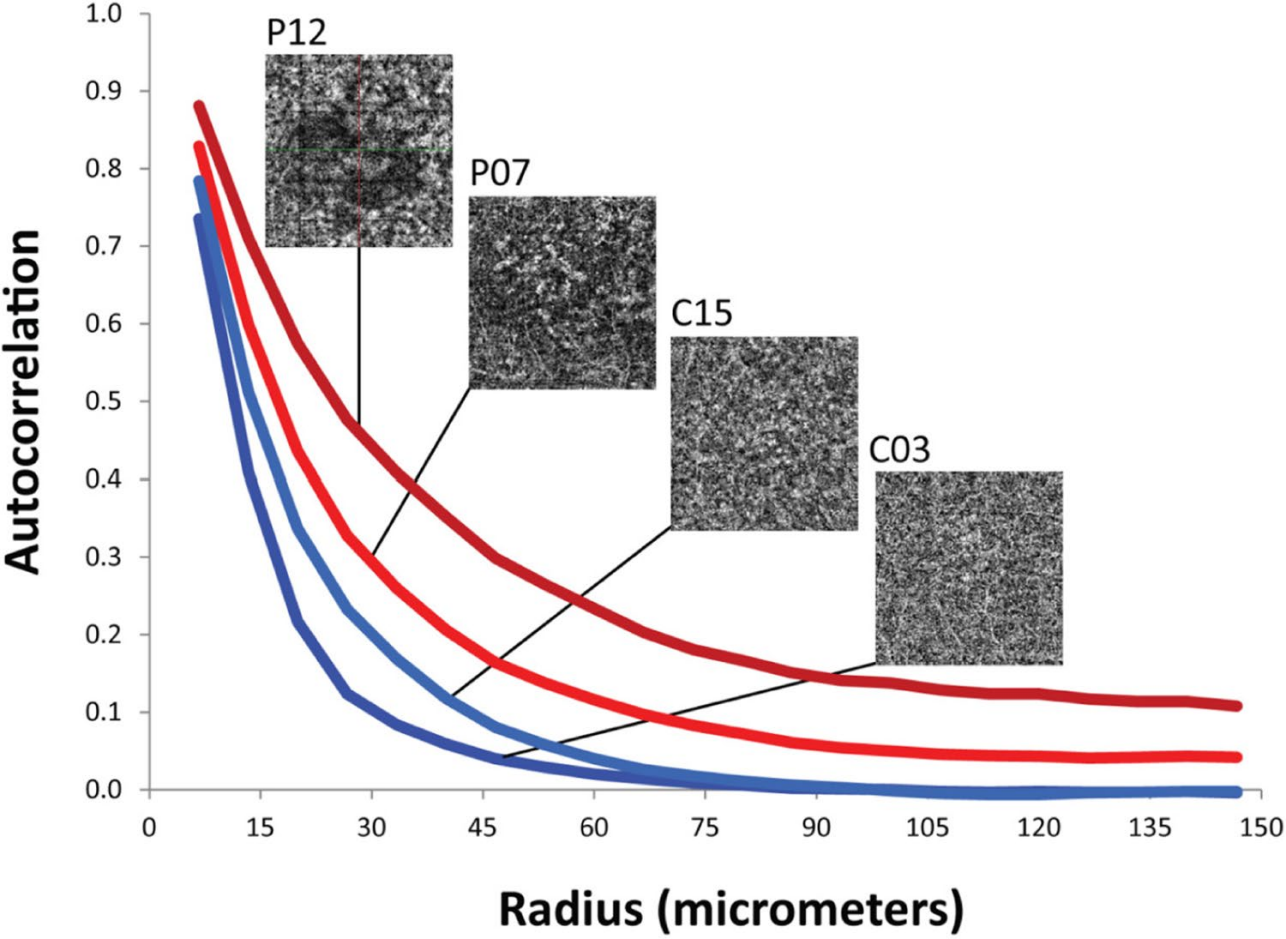
This picture shows the autocorrelation values referring to the different CSC patterns: higher autocorrelation levels of the patients account for a coarser textural pattern, indicative of vascular alterations. The purple line points out the pattern A associated to a very rough texture; the red line indicates the pattern B, a quite gross pattern; the light blue line relates to the pattern D, a quite fine texture; the navy blue line corresponds to the pattern F (the finest texture). The same picture also shows that the alterations involve structures starting from the size of 10 µm, which corresponds to capillaries up to 150 µm in size

Textural analysis was performed using the autocorrelation method, a method commonly used to detect latent, structural patterns within images. Image autocorrelation consists of an evaluation of the correlation between each pair of pixels as a function of the distance (lag) between them. It may be represented by a curve showing the correlation between pixels as a function of distance (Fig. 1). Notably, the correlation, by definition, is not affected by the overall average of the two series of values. This means that autocorrelation is invariant with respect to the relative brightness and contrast of images. This is important, as the image brightness and contrast can be manually adjusted by the operator.

**Fig. 2:**
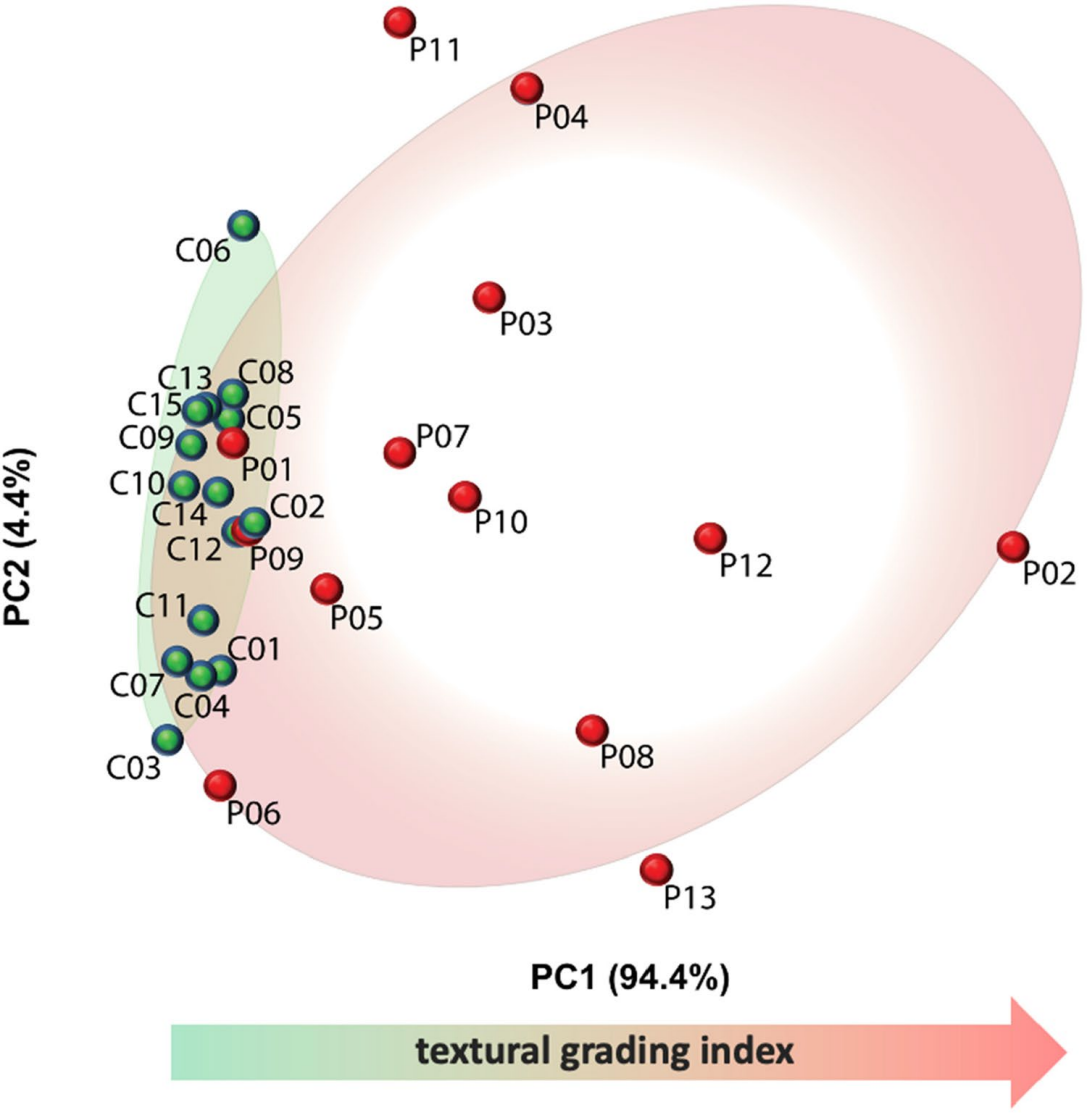
Distribution of OCTA images according to the principal component analysis trendline. The correlation trend is indicated by the red arrow. Red spots represent cases, and green spots represent controls. As it can be seen, all the controls are regrouped all together in correspondence of the low levels of the textural grading index. Whereas, the red points representing the cases are not gathered but they are littered stating very different type of patterns

To measure the CFD of retinal vascular layers, we performed two scans for each eye, and the scan with better signal strength was chosen for analysis. Only images showing serous retinal detachment were analysed (Fig. 2). Images showing subretinal hyperreflective material or pigment epithelial detachment (PED) in the macular area were excluded, as these overlying structures can be associated with shadow artefacts at the level of choriocapillaris OCTA [18–22]. Low-quality OCTA images related to improper centring, movement artefacts, or a signal strength less than 6 were excluded from the analysis. All data were exported to an Excel spreadsheet for statistical analysis. We used a projection artefact removal (Optovue 3D Projection Artefact Removal) in order to optimise all the images.

### Statistical analysis

A descriptive statistical analysis of the quantitative parameters (mean and standard deviation, median, and interquartile range) of the CFD in the superficial plexus, deep plexus, CCL, and outer choroid was performed by one of us (DG) for the CSC patient group and the control group. An independent t-test and chi-squared test were used to compare the two groups. K Cohen analysis was applied to calculate the intra-observer agreement of measurements.

## Results

Forty eyes were diagnosed with CSC (39 patients: 26 males, 13 females; mean age: 49.61 ± 12.5 years) and met the inclusion criteria. BCVA was 0.21 ± 0.17 logMAR (range: 0 ÷ 0.8 logMAR, corresponding to 20/20 ÷ 20/125). 23 eyes (58.9%) had newly diagnosed acute CSC, while 9 eyes (23.07%) had a history of at least 6 months of visual symptoms in the affected eye (chronic form), and 7 eyes (17.9%) were considered affected by recurrent CSC. The control group (7 males, 6 females, mean age: 54.2 ± 12.5 years) had a BCVA of 1.00 logMAR, corresponding to 20/20. In Table 1, patient demographics and clinical characteristics are detailed.

**Table 1:** Demographic characteristics of patients

Cases	26 males; 13 females
Controls	17 males; 16 females
Mean age of cases	Mean age 49.59 ± 12.53
Mean age of controls	Mean age 48.15 ± 12.31
Clinical features	23 acute CSC, 9 chronic CSC, 7 relapsing CSC
BCVA (LogMar)	0.21 ± 0.17 logMAR
Therapies of cases	14 laser therapy; 6 Eplerenone therapy (Inspra 25 mg)
Percentages of patterns	Pattern A: 7.69% — acute CRSC
	Pattern B: 30.76% — acute CRSC
	Pattern D: 13,07% — chronic CRSC
	Pattern E: 38,03% — chronic CRSC
	Pattern F: 10.45%

Demographic characteristics of patients are reported in Table 1.

**Fig. 3:**
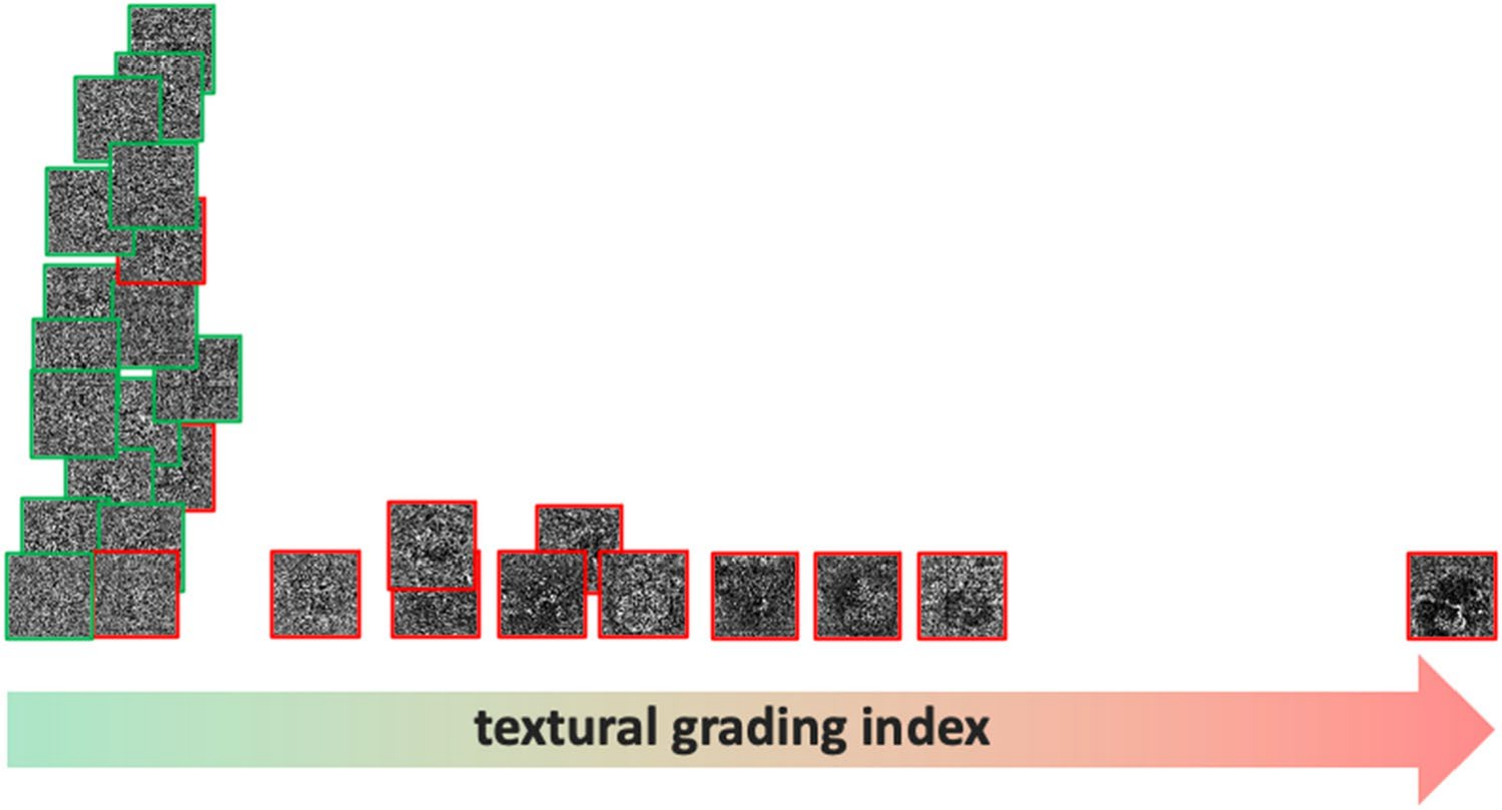
OCTA images arranged horizontally according to the correlation trendline. Here, the correlation trend is indicated by the red arrow. The horizontal arrangement is arbitrary and made solely to avoid the overlap of images. Red pictures represent the cases and the green ones the controls: controls are clustered on the left in correspondence of the lowest texture levels. Case frames become increasingly distant along the highest texture levels

Some examples of OCTA scans of patients and controls utilised in the textural analysis are shown in Fig. 2. Images of patients C06 and P12 exhibit some artefacts, which are likely to be due to eye movement. Four representative autocorrelation curves of two controls and two patients are shown in Fig. 1. In general, the curves of all controls reached the zero value at a distance of 90 μm (equivalent to 18 pixels), indicating a finely homogeneous vascular texture. Conversely, the curves of CSC patients, with the exception of three cases, did not reach the zero value but, instead, plateaued between 0.05 and 0.2 in autocorrelation value, corresponding to ~ 120 μm (24 pixels). However, three patients (P01, P06, and P09) showed curves similar to those of controls. The differences between CSC patients and the controls are already evident in the range of 10 μm. This corresponds to average-sized blood capillaries and is consistent with the presence of microvascular alterations in CSC patients. Over 98% of the information contained in autocorrelation curves, at all lag values, was summarised using principal component analysis (PCA), as shown in Fig. 2. The first principal component (the PCA *x*-axis) accounted for 94.4% of all variance and confirmed a substantial separation between the controls and patients, with the exception of the aforementioned three cases. This suggests that the alignment of subjects (both patients and controls) on the first principal component (Figs. 2 and 3) may be assumed to be a grading of CSC alterations.

On the basis of the different sizes and distributions of dark spots observed in CCL, we identified six vascular patterns (Fig. 1):

Patterns A (7.69%) and B (30.76%) were mainly associated with acute disease; patterns D (13.07%) and E (38.03%) were associated with chronic disease; recurrent disease corresponded to pattern F (10.45%).

Overall, the OCTA features can be summarised by a single value for each subject obtained using PCA (Fig. 2). The higher autocorrelation value of CSC patients accounts for a coarse-texture pattern, indicative of vascular alterations of CCL (Fig. 3). While CSC patients showed variable degrees of texture, all controls disclosed a relatively homogeneous texture (Fig. 3).

The mean subfoveal choroidal thickness for all patients was calculated using the mean value of the B-SCAN of structural SD-OCT included in the Optovue software with respect to age, sex, and dioptres. The mean choroidal thickness was 409.4 ± 94 μm in males and 407.62 ± 91 μm in females. The mean choroidal thickness in the control group was 298.82 ± 78 μm in males and 285.38 ± 71 μm in females. The mean subfoveal choroidal thickness was significantly greater in eyes of patients with CSC than in age-matched normal eyes. The mean subfoveal choroidal thicknesses in images of patterns A, B, C, and E were 407.89 ± 93 (*p* = 0.0003), 408.41 ± 89, 298.77 ± 74, and 398.84 ± 79 μm, respectively.

**Fig. 4:**
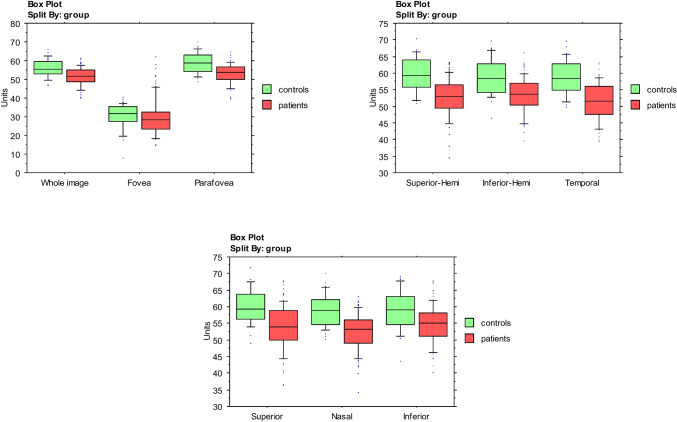
Comparison between patients affected by CSC and healthy individuals

The analysis of CFD in the superficial capillary plexus showed a significant reduction over the whole region (p = 0.023). The superior-hemi and temporal sectors showed a statistically significant reduction in CFD (p = 0.0009 and p = 0.0008, respectively), as shown in Fig. 4 and Tables 2 and 3.

**Table 2:** Capillary flow density: mean and standard deviation in sections of patients with CSC and healthy individuals (independent t-test)

Total confrontation	Patients (N = 41)		Controls (N = 35)		P value
	Mean	SD	Mean	SD	
Whole image	51.39	4.81	55.77	4.81	< 0.0001
Fovea	29.84	10.1	30.44	6.96	0.747
Parafovea	53.02	5.41	58.72	5.41	< 0.0001
Superior-hemi	52.64	6.09	59.48	5.14	< 0.0001
Inferior-hemi	53.3	5.35	58.73	5.44	< 0.0001
Temporal	51.29	5.71	58.52	5.17	< 0.0001
Superior	53.96	6.98	59.93	5.27	< 0.0001
Nasal	52.31	5.58	58.63	5.06	< 0.0001
Inferior	54.38	6.13	58.81	6.04	0.0005
Thickness	338.38	84.57	311.31	20.86	0.061
A	54.15	5.71	58.43	5.37	0.0003
B	46.1	5.11	50.47	4.09	< 0.0001
C	53.97	5.55	58.16	5.76	0.0003

**Table 3:** Capillary flow density: choriocapillary layer, superficial and deep plexuses. Mean and standard deviation in sections of patients with CRSC

Capillary flow density	Choroid		Superficial		Deep	
	Mean	SD	Mean	SD	Mean	SD
Whole image	49.5	13.9	48.99	4.66	53.66	3.76
Fovea	50.6	16	29.37	8.24	30.29	11.68
Parafovea	51.5	14.1	50.71	5.69	55.21	4.1
Superior-hemi	50.5	15.8	50.56	6.43	54.63	5.07
Inferior-hemi	52.6	15.8	50.86	5.26	55.63	4.35
Temporal	51.1	15.7	49.96	5.78	52.57	5.42
Superior	50	18.7	51.14	7.18	56.63	5.68
Nasal	52.3	17.8	50.5	6.03	54.02	4.56
Inferior	53.5	17.5	51.25	5.94	57.34	4.72
Thickness	347	84	338.95	86.24	337.85	84.08
A	46.2	18.2	51.25	5.36	56.9	4.6
B	51.8	13.7	44.91	5.02	47.23	4.99
C	49.5	16.2	50.87	4.71	56.91	4.63

**Figs. 5 and 6:**
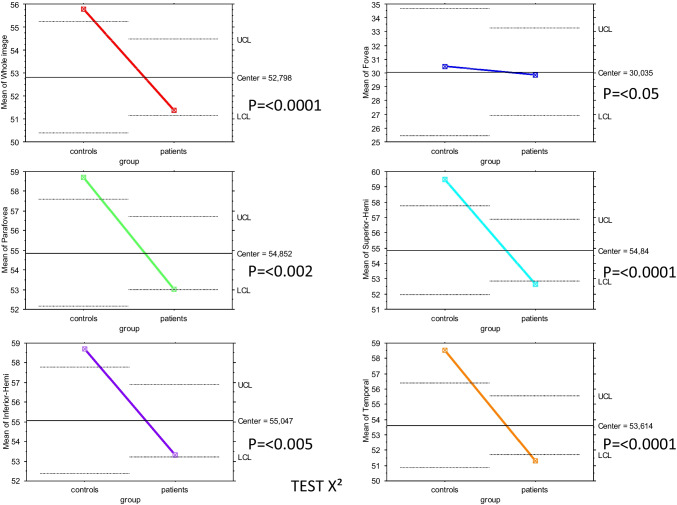

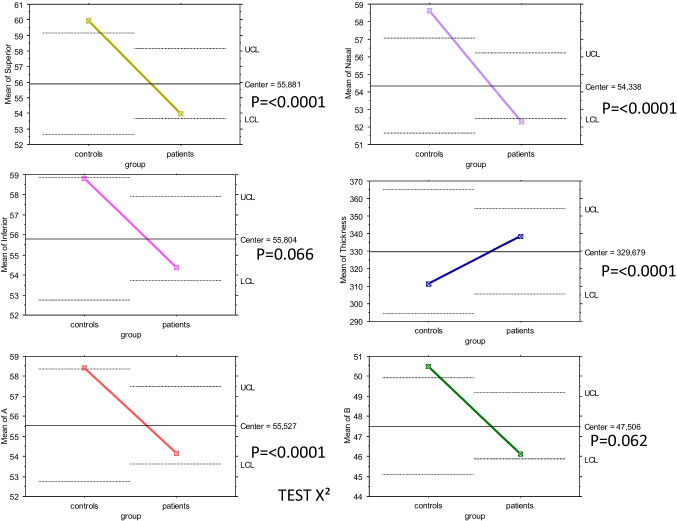
A series of graphics showing the central value between controls and patients with CSC and the position of two groups for each capillary flow density parameter. Comparison was made X^2^ test.

Concerning the deep capillary plexus, the CFD was significantly reduced over the entire image and in the parafoveal, superior-hemi, inferior-hemi, temporal, superior, and nasal sectors (p = 0.0001, Figs. 5 and 6). In the CCL, a statistically significant difference was found in correspondence with the whole image and parafovea, superior-hemi, inferior-hemi, temporal, superior, and nasal sectors (p = 0.0001), (Figs. 5 and 6, Tables 2 and 3). K Cohen of intra-observer measurements was 0.91.

## Discussion

The results of the present study indicate that OCTA scans of the choriocapillaris layer can be processed by quantitative textural analysis to objectively evaluate the disposition of dark areas, i.e. areas of capillary dilatation or areas where blood flow is absent. In this way, it is possible to outline six different vascular patterns, which, interestingly, appear to correlate, with good approximation, with the clinical type of CSC disease. Specifically, patterns A and B correspond to acute forms of disease, patterns D and E are associated with chronic disease, and patterns C and F are associated with control cases. To our knowledge, this is the first study to correlate vascular patterns with the clinical stage of CSC disease.

Undoubtedly, OCTA technology played a fundamental role in obtaining such results. OCTA differs from conventional FA and ICGA in that it generates images of the microvasculature with higher contrast and resolution, mainly because serous leakage, a potential confounding factor, is not recorded.

Previous works performed with multimodal imaging anticipated that dilatation of choroidal vessels, increased permeability, and ischaemic choroidal lobules represent the main features of CSC [23, 24]. Many ICGA studies have described features of abnormal choroidal circulation in CSC, such as dilatation of large choroidal vessels, [23, 24] delayed choroidal artery filling [24, 25], and hyperfluorescence of blurred contours interpreted as choroidal vascular hyperpermeability.[25] Moreover, dark areas have been individuated in the choroid of CSC patients using ICGA examination [26]. Quin et al. postulated that these areas correspond to ischaemic choroidal lobules [27]. This finding was confirmed by Kitaya et al., [2] who correlated the ischaemic regions (detected as areas of decreased signal by ICGA) to a reduction of 45% in the signal detected by laser Doppler flowmetry of the choroid.

In our study, we observed dark spots as single or multiple well-delineated areas in the CCL (Fig. 3). According to previous works using ICGA, the wide black areas observed with OCTA may correspond to a flow void, i.e. ischaemic areas due to focal atrophy of the choriocapillaris, probably secondary to compression by the enlarged vessels from the outer choroid, as previously suggested[28]. Alternatively, the dark zones might be due to the increased diameter of vessels, with consequent hyperpermeability of the CCL, recorded by OCTA as a wide black area of absent signal. As a last hypothesis, dark regions might be produced by light attenuation or altered signals returned by subretinal fluid detachment, flat irregular PED, outer segment photoreceptor elongation, or a combination of these factors, as described by Costanzo et al.[18]

Dark spots always appeared to be associated with a concomitant increase in the reflectivity signal from vessels of the CCL. This finding may be related to the studies of Tittl et al.[29], who found vessel dilatation, with a concomitant increase in subfoveal choroidal blood flow, in chronic-relapsing but inactive CSC, as assessed with laser Doppler flowmetry and ocular perfusion pressure (OPP), and Saito et al.,[30] who evaluated macular choroidal blood flow velocity by laser speckle flowgraphy and found a decrease concurrently with regression of CSC, suggesting the validity of choroidal blood flow elevation in the pathogenesis of acute CSC.

Concerning CFD in CSC patients, we found a statistically significant reduction in flow in all 3 layers examined with respect to the control group. In particular, a reduction in flow in the inner retinal layers was observed, as has been previously reported [30]. Such a lower percentage of capillaries may be the result of ischaemia of the inner neuroretina, in addition to choroidal lobular ischaemia. This reduction in flow may also be due to the autoregulation of the inner blood-retina barrier: in conjunction with the dilatation of CCL capillaries and neuroretinal detachment, neuroretinal flow decreases as a compensatory mechanism to prevent an increase in subretinal fluid levels.

Our study was limited by the retrospective nature of this research, a modest sample size, and a lack of longitudinal follow-up. However, we feel that this study provides a foundation for future investigation into the quantitative pattern of choriocapillaris in CSC, which may represent a valuable tool to refine the classification, to explore the clinical course of the disease, and to implement experimental studies of pattern recognition for the therapeutic response to medical and laser therapy. Moreover, this study confirms a depletion of CFD in correspondence with the inner retinal layers, pointing to an involvement of the inner blood retinal barrier in CSC.

A limitation of this study is the novelty concerning the method of pattern quantification. It is a complex analysis, which requires a high competence level dealing with statistics and image analysis softwares. Hence, we are aware that this is a niche methodology which seeks to contribute a new piece of information in OCT-A study of CSC pathology.

## Data Availability

The authors confirm that all data underlying the findings are fully available without restrictions.
